# Surgery for chronic pyothorax after failed amplatzer closure of bronchopleural fistula

**DOI:** 10.1186/s13019-024-03285-7

**Published:** 2025-01-16

**Authors:** Yohann Vincent, Alessandra Lenzini, Amir Hanna, Nicolas Leymarie, Brendan Le Picault, Elie Fadel

**Affiliations:** 1 Department of Thoracic and Vascular Surgery, Sainte Anne Military Teaching Hospital, 2 Boulevard Sainte Anne, Toulon, France; 2 Department of Thoracic and Vascular Surgery, and Lung Transplantation, Marie-Lannelongue Hospital, Le Plessis-Robinson, France; 3 Pulmonology Department, Marie-Lannelongue Hospital, Le Plessis-Robinson, France; 4 Department of Reconstructive Surgery, Gustave Roussy, Villejuif, France

**Keywords:** Right bronchial fistula, Pneumonectomy, Carina surgery, Amplatzer

## Abstract

**Background:**

Post-pneumonectomy bronchopleural fistula (BPF) is a life-threatening event whose treatment is not standardized.

**Case presentation:**

We report the management of a 28-year-old patient with a 3-year history of BPF complicating right pneumonectomy for congenital emphysema. Despite closure by an Amplatzer device, the patient had chronic pyothorax and severely deteriorated general health and quality of life. An attempt at Amplatzer device removal through an open window thoracostomy failed. A median sternotomy was performed, the carina was resected, and the left main bronchus was anastomosed to the trachea. The thoracostomy was closed using fasciocutaneous pedicled flaps. At 9 months, the patient was doing well.

**Conclusion:**

The utilisation of an Amplatzer device to close a broncho-pleural fistula can have adverse effects and potentially result in fatal sepsis in those cases where it is unsuccessful. The endoscopic treatment of BPF should be reserved for expert centers, considering the location, size, and stability of the fistula when choosing the treatment strategy. The surgical management of failed Amplatzer closure may be extremely complex, requiring a highly experienced team.

## Background

Bronchopleural fistula (BPF) is a life-threatening complication occurring in approximately 15% of pneumonectomy procedures for nonmalignant lung disease. Treatment is not standardized, with options ranging from open window thoracostomy to endoscopic fistula closure and indwelling catheter drainage [1–3]. Endoscopic treatment may be better tolerated than open surgery given the frequently poor general health of patients with postpneumonectomy BPF. Closure can be achieved using a stent, prosthesis, Amplatzer device, or glue. Several case-reports [1–3] and a review article [4] support the efficacy and safety of Amplatzer devices used for BPF closure. However, information is lacking on the management of failed Amplatzer closure.

## Case presentation

The patient was a 28-year-old man referred for chronic pyothorax with sepsis 7 years after right pneumonectomy for congenital pulmonary emphysema and 3 years after the diagnosis of a right BPF treated by endobronchial Amplatzer implantation. He provided signed authorization to report his case. Amplatzer implantation was followed by pyothorax (*Pseudomonas aeruginosa* and *Staphylococcus aureus*) and septic shock requiring chronic chest drainage and indwelling-catheter antibiotic therapy. At the time of referral, he presented with severe sepsis and malnutrition (body mass index of 15.06) despite chronic drainage and multiple courses of antibiotics.

**Fig. 1:**
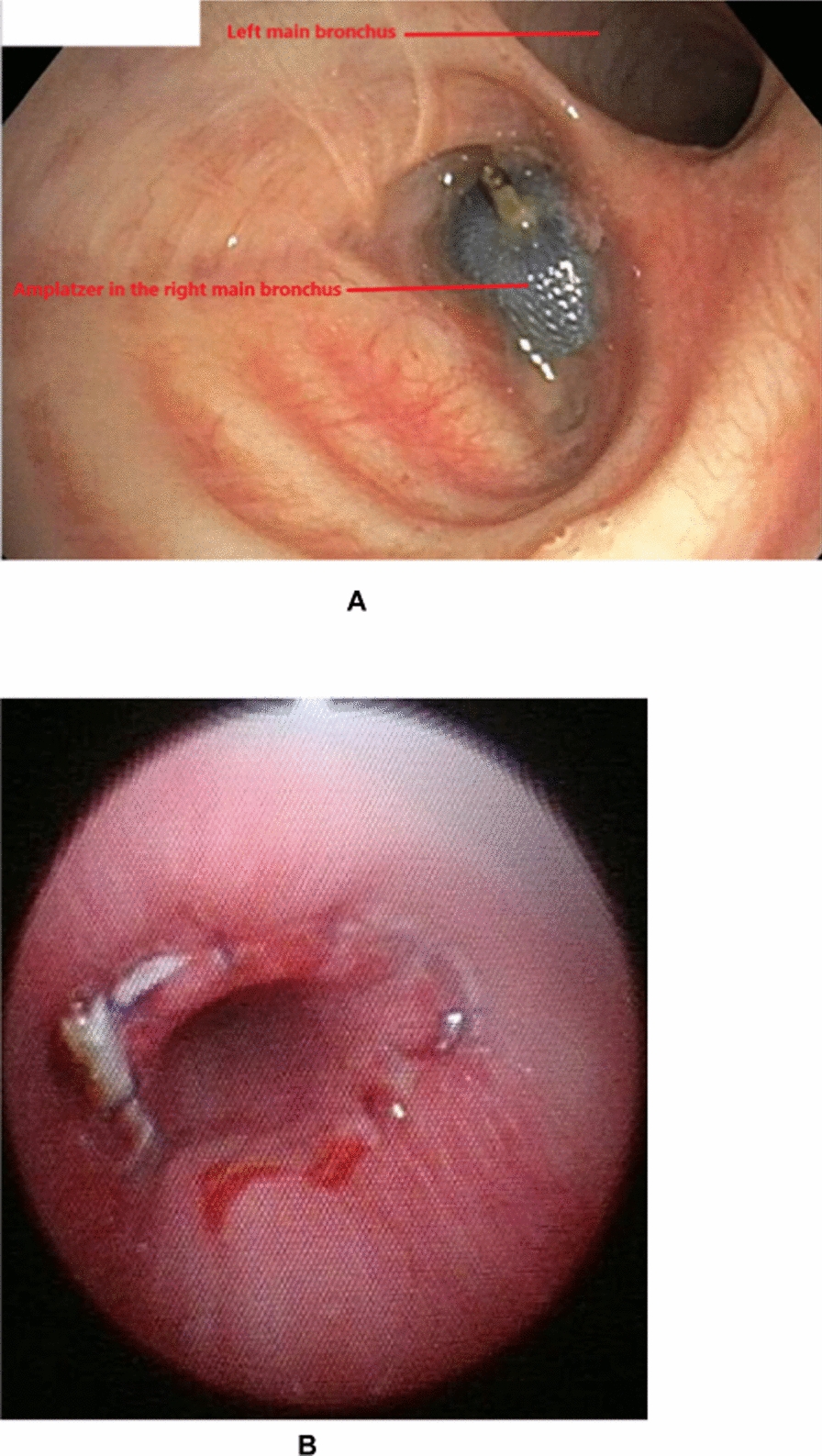
Bronchoscopic appearance: **1A** Before surgery; **2A** After surgery, with the end-to-end anastomosis of the left main bronchus to the trachea

**Fig. 2:**
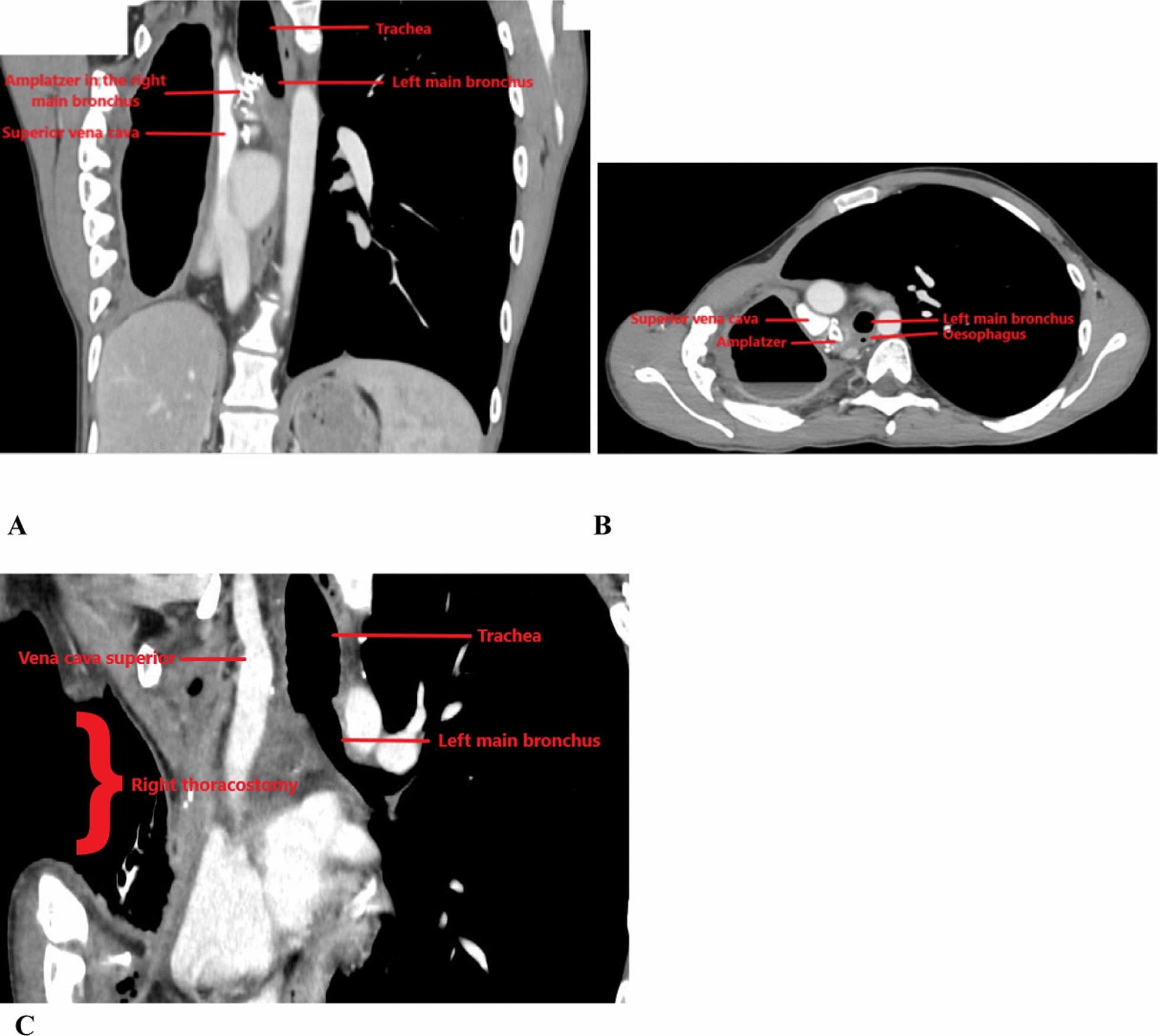
Computed tomography findings: **2A** Coronal view before surgery; **2B** Axial view before surgery; **2C** Coronal view after surgery

Bronchoscopy showed a well-positioned Amplatzer device in the right main bronchus (RMB) with no evidence of bronchopleural fistula (Fig. 1A). By computed tomography, fluid and air were visible in the right pleural cavity. The Amplatzer device was located in close proximity to the right upper lobe vein (RULV) and the right pulmonary artery, suggesting the presence of a bronchovascular fistula (Fig. 2A, B)."

A Clagett thoracotomy was performed and the cavity was cleaned. An attempt to remove the Amplatzer device under endoscopic visual guidance was unsuccessful. The cavity was brushed manually, the patient was turned to the supine position, and a median sternotomy was performed. The trachea was approached between the ascending aorta and superior vena cava (SVC) to avoid opening the right chest cavity. The right main pulmonary artery and RULV were stapled after intrapericardial control. The RMB with the Amplatzer device were dissected free from the SVC and removed without opening the pleural cavity. The carina was resected and the left main bronchus was anastomosed end-to-end to the trachea, with protection by a thymus flap.

**Fig. 3:**
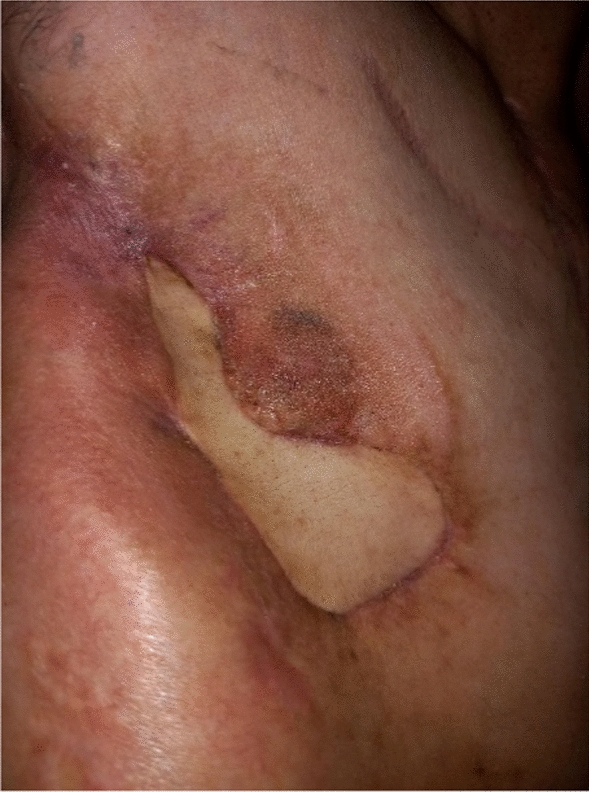
Thoracostomy 1 month after closure using pedicled flaps

The patient was discharged 24 days after surgery. However, over the first 3 postoperative months repeated debridement of the right pleural cavity and antibiotic treatments were required. Refeeding was via a jejunostomy tube. The clinical, fiberoptic bronchoscopy (Fig. 1B), and computed tomography findings (Fig. 2C) improved. Six months after surgery, the thoracostomy was closed using right dorsalis major and serratus major fasciocutaneous pedicled flaps (Fig. 3). The jejunostomy tube was removed. Three months later, the trachea was fully healed and the patient was in good general health with no evidence of infection.

## Discussion and conclusions

We report the successful open surgical treatment of persistent infection after failed Amplatzer closure of a postpneumonectomy BPF. Importantly, both the general health and quality of life in this young patient were severely impaired by the chronic infection. Tight adhesions precluded endoscopic removal of the Amplatzer device. A major surgical procedure including carinal resection, transpericardial division of the right pulmonary artery and RULV, and SVC release was required. The outcome was favorable.

Postpneumonectomy BPF represents a serious complication, with a reported mortality rate of up to 22% [5]. Open surgical closure is the reference standard treatment. However, the severe deterioration in general health caused by BPF makes many patients poor candidates for surgery. Endoscopic implantation of an Amplatzer device has therefore been suggested as an alternative. Although most publications are case-reports, [1, 2] a 2014 study described a cohort of 31 patients with a mean follow-up of 17.6 months [4]. Mean age was 66.7 ± 17.1 years and the reason for lung resection was cancer in 29/31 patients. Either an Amplatzer occluder or an Amplatzer vascular plug was used depending on the anatomical configuration. Implantation was successful with resolution of fistula-related symptoms in 30/31 patients. All 4 early deaths and 6 of the 12 subsequent deaths were due to sepsis. Another study included 13 patients, 7 of whom had cancer and 6 had tuberculosis [3]. The device became dislodged in 3 patients but outcomes were favorable in the remaining 10 patients, although follow-up duration is not reported. Device displacement may occur due to progression of the fistula, notably if closure is done before the fistula is fully formed or if the patient has progressive tuberculosis. Dislodgement is more likely to occur if the fistula is large, and accurate fistula size measurement is crucial when choosing the treatment strategy. In both studies, evidence was obtained that the device was gradually overgrown by granulation tissue [3, 4].

The transternal-transpericardial approach represents a valuable alternative in select cases of postpneumonectomy BPF. This technique allows surgeons to avoid the infected pleural cavity, thereby reducing the risk of intraoperative contamination and postoperative complications such as reinfection or sepsis. In our patient, this approach proved advantageous given the extensive adhesions and the persistent infection within the pleural space. By utilizing this route, we successfully achieved fistula closure while maintaining a clean surgical field, facilitating more precise handling of major structures such as the pulmonary artery, superior vena cava, and the carina.

To our knowledge, no prior data exist on the management of failed Amplatzer BPF closure complicated by persistent infection and/or fistulation into a pulmonary artery or vein. These complications can cause fatal sepsis. Endoscopic treatment of BPF should be performed exclusively in expert centers. The location, size, and stability of the fistula are important to consider when choosing the treatment strategy. In our patient, the excessive size of the fistula may have contributed to the failure of the Amplatzer device. Surgical management of failed Amplatzer closure may be exceedingly complex and necessitates a highly experienced multidisciplinary team.

## Data Availability

No datasets were generated or analysed during the current study.

## Abbreviations

BPF: Bronchopleural fistula
RMB: Right main bronchus
RULV: Right upper lobe vein
SVC: Superior vena cava
